# 24-Month outcomes of gonioscopy-assisted transluminal trabeculotomy for congenital glaucoma

**DOI:** 10.5935/0004-2749.2024-0309

**Published:** 2025-09-10

**Authors:** Maria Betânia Calzavara Lemos, Bruno Mendes de Faria, Mariana Botrel Cunha, Frederico de Miranda Cordeiro, Pedro Hélio Estevam Ribeiro Júnior, Ana Luiza Bassoli Scoralick, Fábio Bernardi Daga, Fabio Nishimura Kanadani, Tiago Santos Prata

**Affiliations:** 1 Glaucoma Unit, Hospital Medicina dos Olhos, São Paulo, SP, Brazil; 2 Hospital Universitário Onofre Lopes, Natal, RN, Brazil; 3 Universidade Federal de São Paulo, São Paulo, SP, Brazil; 4 Ver Excelencia em Oftalmologia, Goiânia, GO, Brazil; 5 Mayo Clinic, Jacksonville, FL, USA

**Keywords:** Glaucoma, Open-angle/surgery, Gonioscopy, Trabeculectomy/methods, Intraocular pressure, Antihypertensive agents/therapeutic use

## Abstract

**Purpose:**

To report the surgical outcomes of patients with primary congenital glaucoma who underwent gonioscopy-assisted transluminal trabeculotomy.

**Methods:**

This retrospective, noncomparative, interventional study included consecutive patients with primary congenital glaucoma with uncontrolled intraocular pressure undergoing gonioscopy-assisted transluminal trabeculotomy between January 2017 and January 2020. The included participants were followed up for at least 24 months, and only one surgeon performed all the procedures. The number of glaucoma medications, pre- and postoperative intraocular pressure, treatment extension (in quadrants), surgical complications, and any associated events or interventions were documented.

**Results:**

This study included 13 eyes from 10 patients (mean age, 4.5 ± 3.2 years; range, 3 months to 10 years). After a 24-month follow-up, the mean intraocular pressure significantly decreased from 26.1 ± 3.7 to 11.8 ± 2.5 mmHg (p<0.001). The mean number of glaucoma medications was reduced from 3.3 ± 0.5 to 0.85 ± 1.0 (p<0.001). At the end of the follow-up interval, all eyes (13 out of 13) had an intraocular pressure between 7 and 15 mmHg. In 11 of 13 eyes (84.6%), gonioscopy-assisted transluminal trabeculotomy was performed in all quadrants (360º). The most frequent postoperative complication was transitory (self-limited) hyphema (7 out of 13 eyes [53.8%]). No sight-threatening adverse events occurred during the entire follow-up period.

**Conclusions:**

The 2-year follow-up results indicated gonioscopy-assisted transluminal trabeculotomy as an efficient and safe option for primary congenital glaucoma treatment with minimal postoperative complications.

## INTRODUCTION

Primary congenital glaucoma (PCG) is a rare pathological condition affecting approximately 1 in every 10,000 children in the first year of life^(1)^. It is considered as one of the prevalent childhood eye diseases that pose potential risk for irreversible blindness if not promptly recognized and adequately managed^(2)^. The pathophysiology of PCG involves the anomalous development of the anterior chamber angle and trabecular meshwork, culminating in increased resistance to aqueous humor outflow and high intraocular pressure (IOP) elevation^(3)^. Other main ocular findings included buphthalmos, corneal edema, epiphora, and photophobia^(4)^.

The World Glaucoma Association Consensus^(5)^ states that childhood glaucoma is characterized by IOP-related eye damage. Unlike adult glaucoma, in which the diagnosis often focuses on optic nerve damage, in childhood glaucoma, the definition encompasses the impact of elevated IOP on various ocular structures during infancy. Ocular enlargement, Haab striae, and increased cup-to-disc ratio are important indicators, sometimes more so than IOP measurements alone. As a subset of primary childhood glaucoma, PCG involves isolated angle anomalies with or without mild congenital iris anomalies. It satisfies the criteria for glaucoma diagnosis and typically presents with ocular enlargement. Subcategories based on age of onset include neonatal or newborn onset (0–1 month), infantile onset (>1–24 months), and late onset or late recognition (>2 years). In rare cases, spontaneously arrested PCG occurs, where normal IOP is observed, but typical signs of PCG are present.

Early surgical treatment is crucial to achieving good visual prognosis. The gold-standard procedures are goniotomy and trabeculotomy. The surgical choice between these approaches rely on the patient’s clinical condition, individual factors (e.g., child’s age, degree of ocular malformation, corneal transparency, and axial length), and surgeon’s experience^(2)^. Both procedures are based on the removal of the residual mesodermal tissue (trabecular membrane persistence), improving the aqueous humor outflow^(6^,^7)^. In case of surgical failure, standard trabeculectomy or glaucoma drainage implants may be considered.

The trabecular meshwork’s mechanical split to decrease the aqueous flow’s resistance is not an innovation, such as *ab-externo* trabeculotomy that has been utilized for decades as a surgical alternative for cases of open-angle glaucoma^(8^,^9)^. However, *ab-interno* trabeculotomy has recently emerged as a less-invasive alternative owing to its capacity to preserve the sclera and conjunctiva from manipulation. Gonioscopy-assisted transluminal trabeculotomy (GATT) is an *ab-interno* trabeculotomy surgery reported by Grover et al.; it was developed as a surgical alternative for the management of open-angle glaucoma^(10^-^12)^. Good results, including decreased IOP and number of glaucoma medications, have been described^(10^-^12)^. Although a relatively large number of GATT studies involving adults have been conducted, there are scant data on its results in PCG. The present study aimed to describe a series of patients with PCG subjected to GATT.

## METHODS

### Ethical considerations

Our study adhered to the principles of the Declaration of Helsinki and was submitted and approved by the ethics committee of *Hospital Onofre Lopes*.

### Patient and data collection

This retrospective, noncomparative, interventional study included consecutive patients with PCG aged <14 years with uncontrolled IOP who underwent GATT between January 2017 and January 2020. PCG was diagnosed in accordance with the World Glaucoma Association Consensus^(5)^ criteria, as described in the Introduction section. Eyes with secondary glaucoma, peripheral anterior synechiae (PAS) ≥90°, hazy cornea, or GATT <90° were excluded. The same experienced surgeon performed all the surgeries at the *Hospital Universitário Onofre Lopes*, Rio Grande do Norte, Brazil.

Available visual acuity data were collected from the patients’ medical records. As a routine, visual acuity was assessed using the conventional Snellen chart in children who were literate and familiar with the alphabet. For nonliterate children or those not yet familiar with the alphabet, alternative visual acuity tests were employed, including the Tumbling E chart and an adapted Lea symbols chart (simple figures are presented). For participants with impaired vision, visual acuity was measured through finger counting (at varying distances), hand motion, light perception, or no light perception (no light perception from a bright flashlight). Electrophysiological test was not employed for visual function assessment.

Medically uncontrolled subjects were identified as those with IOP beyond the threshold established considering the extension of structural and/or functional loss, risk factors, and age^(13)^. The numbers of antiglaucoma medications as well as pre- and postope rative medications, IOP measurements, treatment extension (in quadrants), successive procedures, and surgical complications were documented. The median of two preoperative IOP values obtained in different days was used as the baseline IOP. Only subjects who were followed up for at least 24 months were enrolled. In cooperative patients older than 3 years, IOP measurement was standardized via Goldmann applanation tonometry in the outpatient clinic. In those younger than 3 years, or any uncooperative child, the routine included IOP measurement in the surgical center under anesthesia using the Perkins tonometer.

The major study outcomes were the magnitude of IOP reduction and success rates at 24 months of follow-up. Success was defined as achieving a final IOP between 7 and 15 mmHg (adapted from the World Glaucoma Association Guidelines)^(14)^. An IOP of 15 mmHg or lower in patients with PCG indicates better control of optic nerve damage progression and reduced need for additional therapies. Moreover, a postoperative IOP above 7 mmHg indicates low risk for ocular hypotony and hypotonic maculopathy^(14^,^15)^. Surgical failure was defined as an IOP above 15 mmHg in two successive postoperative appointments, the occurrence of light perception loss, or the need for another reoperation to reduce the IOP.

### Surgical procedure

**Figure 1:**
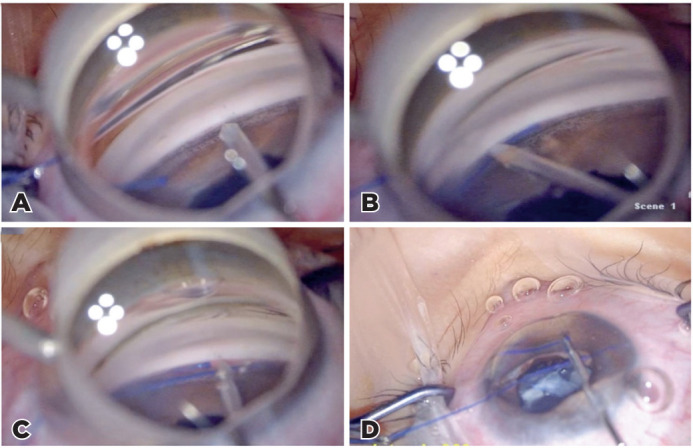
Gonioscopy-assisted transluminal trabeculotomy. (A) Creation of goniotomy incision on the nasal trabecular meshwork using a 23-gauge needle. (B) A blunt-end 5.0 polypropylene suture is introduced through the goniotomy cleft into Schlemm’s canal using a microsurgical forceps. (C) The distal blunt-end of the suture is visualized. (D) While the distal end of the suture is held using a microsurgical forceps, traction is placed on the proximal end, creating a trabeculotomy 360°.

A single-center surgeon (B.M.F.) performed all surgeries in a standardized manner^(16)^. Surgery began with superior temporal and nasal corneal paracentesis and continued with carbachol and lidocaine injection in the anterior chamber, which was then filled with 2% methylcellulose. Subsequently, a nasal goniotomy incision was made using a 26-gauge needle, and then a blunt-end 5-0 polypropylene suture was introduced into the goniotomy cleft, permeated the entire circumference reaching the initial goniotomy site with the support of a 23-gauge microsurgical forceps’ tip (Figure 1). Then, the suture was removed, which resulted in a circumferential trabeculotomy. When anatomical resistance was detected, a new goniotomy incision was made to achieve the suture’s head, which resulted in trabeculotomies ranging from 90° to 360º. Methylcellulose was injected occasionally to control hyphema and hypotony and then flushed from the anterior chamber via irrigation with a balanced salt solution. The amount of residual methylcellulose was determined according to the degree of blood reflux in the episcleral veins^(12^,^16)^. The postoperative prescription was the same for all patients: topical pilocarpine 2% (2×/day for 2 weeks), topical moxifloxacin (4×/day for 1 week), and topical prednisolone 1% (4 weeks in weaning). The management of topical hypotensive drugs was individualized according to each patient’s needs.

### Statistical analysis

Clinical and demographic variables were evaluated using descriptive statistics.

Based on data normality, the Wilcoxon or paired *t-*test was employed to analyze and compare the baseline and postoperative parameters. Computerized statistical analysis was conducted using the MedCalc software (MedCalc Inc., Mariakerke, Belgium). The critical p-value was set to 0.05.

## RESULTS

This study included 13 eyes from 10 patients (mean age, 4.5 ± 3.2 years; range, 3 months to 10 years). Approximately 60% of the patients were women, and 77% (10 out of 13) of the eyes had previously undergone glaucoma surgery: trabeculotomy, n=7; goniotomy, n=1; trabeculectomy, n=1; and trabeculotomy plus trabeculectomy, n=1. Table 1 presents the detailed demographic and ocular characteristics of the patients.

**Table 1:** Demographic and ocular characteristics of the patients

Parameters	Study Patients
Age (mean ± SD), years	4.5 ± 3.2
Female sex	6 (60%)
Previous glaucoma surgery	10 (77%)
Trabeculotomy	7 (53.8%)
Goniotomy	1 (7.7%)
Trabeculectomy	1 (7.7%)
Trabeculotomy plus trabeculectomy	1 (7.7%)
Preoperative IOP (mean ± SD), mmHg	26.1 ± 3.7
Preoperative topical hypotensive drugs (mean ± SD)	3.3 ± 0.5
Postoperative IOP (mean ± SD), mmHg	11.8 ± 2.5
Postoperative topical hypotensive drugs (mean ± SD)	0.8 ± 0.9
Postoperative hyphema	7 (53.8%)
Hyphema resolution (mean ± SD), days	5.8 ± 1.9

SD= standard deviation; IOP= intraocular pressure.

**Figure 2:**
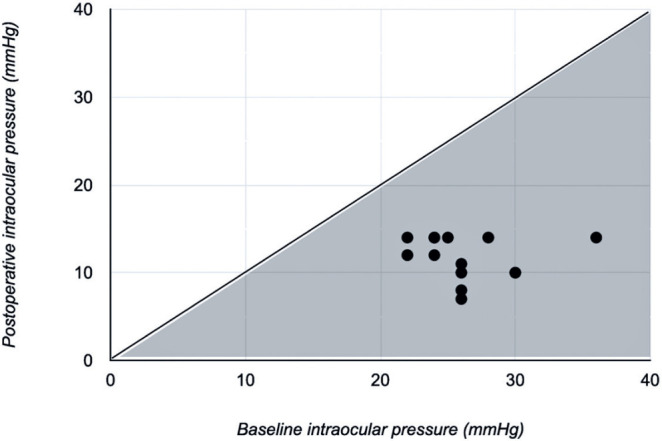
Scatter plot of the intraocular pressure results before and after treatment. Eyes below the solid line (gray area) had lower intraocular pressure values at the last follow-up visit (month 24) compared with the baseline.

After 24 months of follow-up, the mean IOP significantly decreased from 26.1 ± 3.7 to 11.8 ± 2.5 mmHg (p<0.001). Figure 2 demonstrates that all patients experienced postoperative IOP reduction, but the magnitude of the reduction was not significantly correlated with the preoperative IOP (r=−0.02; p=0.94).

The mean number of topical hypotensive drugs decreased from 3.3 ± 0.5 to 0.85 ± 1.0 (p<0.001). At the end of the follow-up period, all eyes had an IOP between 7 and 15 mmHg. GATT was performed in all quadrants (360º) in 11 of the 13 eyes (84.6%), in only 1 quadrant (90º) in 1 eye, and in 3 quadrants in the other eye.

The most frequent postoperative complication was transitory (self-limited) hyphema (7 out of 13 [53.8%]). In all cases, complete hyphema resolution was observed in the first postoperative week. In terms of visual status, all participants had visual acuity of light perception or better at study entry. During the entire follow-up period, no sight-threatening event occurred, and light perception loss was not reported.

## DISCUSSION

Trabeculotomy and goniotomy are considered as the gold-standard surgical procedures for PCG treatment^(17^,^18)^. However, surgeons and researchers have been constantly seeking more effective but safer and less-invasive proceadures for PCG management. In this context, through an investigation of the midterm GATT outcomes in patients with PCG, we have documented not only high success rates but also a relatively favorable safety profile, with no serious sight-threatening adverse events. Most patients in our study had previously undergone glaucoma surgery. In addition to the strong surgical expertise of the involved surgeons, there is robust prior scientific evidence from studies that have assessed the performance of GATT in patients with a previous history of glaucoma surgeries—both in adults and children—demonstrating favorable outcomes in terms of efficacy and safety^(19^,^20)^.

The key findings of our study were efficacy and safety profile. Despite the low number of studies evaluating GATT outcomes in patients with PCG, we have observed a considerable increase in the number of publications in recent years^(21^-^26)^. In our study, the overall mean IOP reduction after 24 months of follow-up was 54.8%, which is comparable to those in other studies (37.1% to 77.7%)^(11^,^27)^. In addition, case reports noted IOP reduction from 41.2% to 68.4% after GATT in patients with secondary congenital glaucoma^(28^,^29)^. In our study, a success rate of 100% was achieved during the 2-year follow-up. This result contrasts the 33.3% success rate reported by Quan et al. in their study involving 21 patients with PCG who underwent GATT^(30)^. Their study demonstrated that for every increase in age at the time of surgery of 5 years, the risk of GATT failure decreased by 37%. Thus, the patient’s median age in our study was approximately 5 years, which may have influenced the success rate. Aside from the mean age, we also believe that the differences observed between our study and that of Quan et al. were due to the follow-up period (24 months in our study and up to 75 months in Quan et al.’s study).

It is essential to review our major clinical outcomes. As previously mentioned, a few studies have focused on GATT results in PCG, and it is well known that there is no definitive treatment for most PCG cases. Consequently, many patients will undergo multiple procedures during their lifetime. In this context, a less invasive and equally efficient procedure, particularly if angle-based and conjunctival sparing, could be a promising alternative. The fact that GATT yielded good results in different forms of open-angle glaucoma makes it a good candidate for PCG management. Our 2-year follow-up results indicated an efficient IOP reduction, with a positive impact on medication burden, suggesting that GATT is a suitable alternative to gain time during the treatment course of these patients. Although still based on a small number of cases, the procedure yielded good outcomes even in eyes that previously underwent glaucoma surgeries. Interestingly, similar findings have been reported in adults^(31)^. Considering that we are usually dealing with very young patients with refractory disease, performing GATT in those with a previous history of surgery seems promising. In addition, in terms of possible technical difficulties, we did not observe any major surgical challenge in these eyes (clear cornea and less than 90° of PAS are mandatory preoperative requirements) as it was possible to treat the entire angle length in more than 80% of the patients, which is consistent with the literature^(32)^. Finally, in addition to the effectiveness of GATT already presented in other studies and corroborated in our study, its safety should also be reported, given that few complications have been reported in the literature^(32)^.

Some specific limitations need to be highlighted while interpreting the results of our study. First, our results are limited due to the relatively small sample size and retrospective design of the study. Owing to the study’s retrospective nature, some parameters were unavailable in the patients’ medical charts, such as axial length and structural/functional tests. Therefore, we were unable to investigate disease progression overtime. Second, as all procedures were performed by the same experienced surgeon, our findings need to be confirmed by other surgeons. Third, our study was not sufficiently powered to explore possible success predictors. Finally, future studies with longer follow-up periods are warranted to access long-term success rates and complications.

In conclusion, our findings indicate that GATT is an efficient option for PCG treatment with minimal postoperative complications. Further studies involving a larger sample and longer follow-up are needed to better investigate the procedure’s long-term efficacy and durability.

## Data Availability

The datasets generated during and/or analyzed during the current study are available on demand from referees.
